# Structure-Dependent Parameter Trade-Off Optimization on *R*_on_*C*_off_ and Power Compression of AlGaN/GaN HEMTs for RF Switch Application

**DOI:** 10.3390/mi17020163

**Published:** 2026-01-27

**Authors:** Xu Zou, Meng Zhang, Ling Yang, Bin Hou, Mei Wu, Chupeng Yi, Hao Lu, Mao Jia, Qian Yu, Yutong Jiang, Xiaohua Ma, Yue Hao

**Affiliations:** State Key Discipline Laboratory of Wide Band-Gap Semiconductor Technology, School of Microelectronics, Xidian University, Xi’an 710071, China; zouxu1714@163.com (X.Z.); houbinme@163.com (B.H.); wumei_xdu@163.com (M.W.); yicp905@163.com (C.Y.); luhao@xidian.edu.cn (H.L.); jm@stu.xidian.edu.cn (M.J.); 18829036159@163.com (Q.Y.); 20009100586@stu.xidian.edu.cn (Y.J.); xhma@xidian.edu.cn (X.M.); yhao@xidian.edu.cn (Y.H.)

**Keywords:** high electron mobility transistor, radio frequency switch, small-signal performance, *R*_on_ *C*_off_, power compression performance, device structure

## Abstract

This paper presents, for the first time, the structure-dependent parameter trade-off optimization on figure-of-merit (*R*_on_*C*_off_) and power compression of AlGaN/GaN high electron mobility transistors (HEMTs) for radio frequency (RF) switch applications. For GaN HEMTs operating in switching mode, it was demonstrated that *R*_on_*C*_off_ can be effectively reduced by increasing the gate foot length (*L*_g_foot_), decreasing the gate cap length (*L*_g_cap_), reducing the gate bias resistance (*r*_g_), and adopting a high work function metal for the gate electrode (*Φ*_g_). However, these parameter adjustments affect power compression and *R*_on_*C*_off_ in opposing manners. This paper also presents supplementary research on the effects of source-drain spacing (*L*_ds_) and gate width (*W*_g_) on switching performance. This research achieves a dynamic balancing method for structural parameters, delivering application-specific design rules for different scenarios ranging from high-frequency to high-power applications.

## 1. Introduction

As an important part of RF front-end modules, RF switches are used in scenarios such as radar, base stations, and satellites and have always been popular for research. High-performance RF switches need low insertion loss (IL), high isolation (ISO), high power handling capability, and wide bandwidth.

Compared to microelectromechanical systems (MEMS) [1], PIN diode switches [2], and GaAs HEMTs [3], GaN HEMTs are gradually becoming the mainstream of high-power RF module applications due to their low power consumption, high RF performance, and easy integration in MMICs [4]. In the field of GaN-based RF switches, recent studies focused mainly on the direction of GaN-based RF switch MMIC circuits, usually based on the limited GaN process line scheme, by changing different circuit topologies to enhance the performance of GaN RF switches [5,6]; however, there are still relatively few studies on the influence of the device structure on the RF transmission performance and power handling of GaN HEMTs [7,8]. While numerous studies have focused on optimizing individual performance metrics, they have not systematically revealed the complex trade-off relationships that exist among different structural parameters, as well as between these parameters and multiple performance indicators. RF switches’ operating frequency and power compression capability are still the core challenges in this field.

This paper investigates the influence of device geometries, *r*_g_, and *Φ*_g_ on *R*_on_*C*_off_ and power compression of AlGaN/GaN HEMTs used as RF switch devices.

## 2. Device Structure and Fabrication

**Figure 1:**
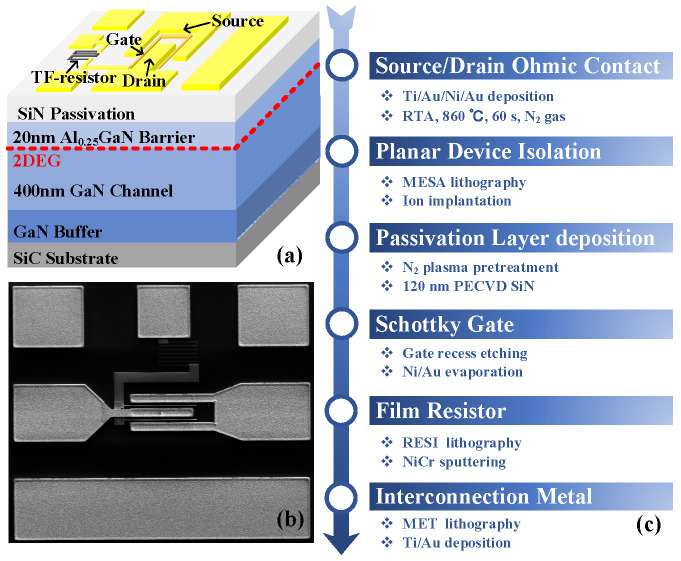
(**a**) The schematic cross-sectional structure of the GaN-on-SiC HEMTs used for RF switches. (**b**) The electron microscopic diagram of the GaN-on-SiC HEMTs used for RF switches. (**c**) Process flow for the fabricated GaN-based HEMTs in this work.

The conventional AlGaN/GaN HEMT for RF switch application was fabricated on the epitaxial structure, which was grown on substrate by metal–organic chemical vapor deposition (MOCVD) and consisted of an AlN nuclear layer, a GaN buffer layer, a GaN channel layer, and an AlGaN barrier layer from bottom to top. The 2DEG sheet density of the epitaxial material at room temperature was 1.071 × 10^13^ cm^−2^, with a sheet resistance of 333 Ω/sq. The device process was started by source and drain ohmic formation, including Ti/Al/Ni/Au metal stack evaporation and rapid annealing at *N*_2_ atmosphere at 860 °C for 60 s. After multilayer N+ injection to isolate the device, a 120 nm SiN passivation layer was deposited. A gate window was identified by photolithography and CF4-based plasma etching, and a T-shaped gate profile was realized using a Ni/Au or W/Au metal stack. A 30 nm NiCr thin film resistor was formed by sputtering. Finally, Ti/Au metal stack interconnections of the device were realized. The fabricated GaN HEMT RF switch device is shown in detail in Figure 1a–c.

Test structure layouts were specifically set for the characterization of RF switch. The device structure of the switch was symmetric *(L*_gs_ = *L*_gd_) with a Schottky contact gate between the source and drain [9]. The ground-signal-ground (GSG) pads on both sides of the device serve as standard RF probe test interfaces, ensuring impedance matching and signal integrity during on-wafer high-frequency measurements. The independent gate bias pad is physically separated from the RF GSG structure, providing a DC feed path for the static gate voltage and preventing mutual interference between the RF signal and the DC bias circuit. A large resistor was added between the gate’s DC pad and the HEMT’s gate. The gate length is 500 nm, which is constrained by the lithography tool’s resolution limit to ensure process robustness. This choice does not hinder the clear and effective investigation of the core physical mechanisms mentioned above.

## 3. Measurement Results and Analysis

This paper pays attention to the mechanisms of how the dimensions and key processes of RF switch devices impact their small-signal and power characteristics, focusing on different *L*_ds_, *L*_g_foot_, *L*_g_cap_, *W*_g_, *r*_g_, and *Φ*_g_. Specific parameter settings and variables are detailed in Table 1.

**Figure 2:**
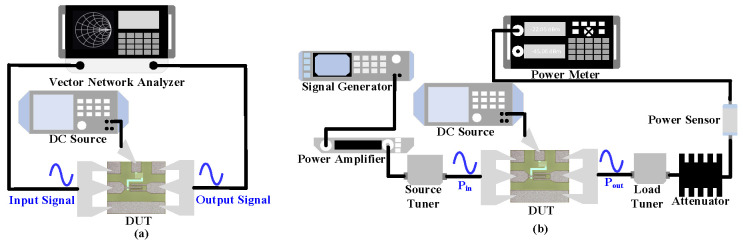
(**a**) Schematic of the small signal measurement setup for RF switch devices. (**b**) Schematic of the power measurement setup for RF switch devices.

This paper characterizes IL in the on-state and ISO in the off-state of the RF switch device through small signal testing. The test equipment setup is shown in Figure 2a, with the vector network analyzer (Agilent E8363B, Agilent Technologies, Santa Rosa, CA, USA) providing a small signal, which enters the source/drain terminals of the RF switch device. After selection, it is output from the drain/source terminals and finally enters the vector network analyzer to obtain S-parameters for analysis. Additionally, the RF switch device’s gate is externally connected to a DC source meter (Keysight B2902B, Keysight Technologies, Penang, Malaysia) to obtain the DC control bias.

Power compression refers to the phenomenon where the output power of an amplifier decreases as the input power exceeds a certain point. This phenomenon arises from nonlinearity in the device’s transfer characteristics, leading to a reduction in gain and output power above specific input levels.

This paper characterizes the power compression capability of the RF switch device in the on-state through load-pull testing. The construction of the test platform is shown in Figure 2b. The signal is generated by the signal source (Agilent E8257D), amplified by the driver amplifier (OPHIR5192, OPHIR Optronics Ltd., Jerusalem, Israel) to obtain a large signal. The signal passes through the RF switch device, and the output power is finally collected by the power probe and displayed by the power meter (Agilent N1912A). Additionally, the source/load tuner in the test system provides a fixed 50 Ω input and output impedance for the device under test, and the DC source meter Keysight B2902B provides gate bias [10]. During testing, large signals enter from the source/drain of the RF switch device and exit from the drain/source.

The simulation framework defines a physics-based model for simulating AlGaN/GaN HEMTs. The Shockley–Read–Hall (SRH) recombination model is enabled to capture the dominant carrier recombination mechanism via trap levels within the bandgap. A critical part of the model for GaN-based HEMTs involves the calculation of polarization charges. The simulator computes the strain within the heterostructure and, based on this strain and the material’s piezoelectric coefficients, derives the piezoelectric polarization charge. This is coupled with the inherent spontaneous polarization charge.

### 3.1. Source-Drain Spacing

**Figure 3:**
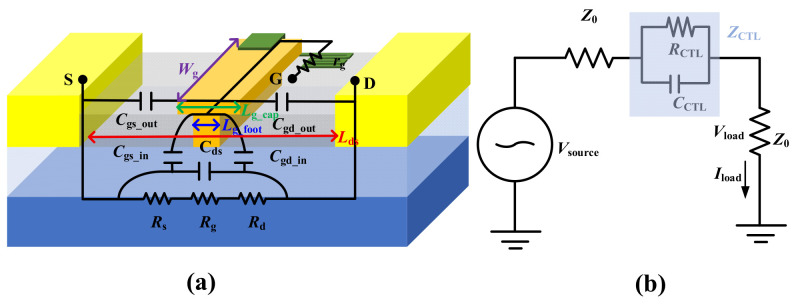
(**a**) Equivalent circuit of GaN HEMT for RF switch application. (**b**) Series-connected ideal control element in a circuit.

Figure 3 shows the equivalent circuit of the switch device, which is composed of a series of intrinsic parameters. The small-signal performance of the switch device is analyzed starting from these parameters. IL or ISO is shown below [11]:

(1) $$\begin{array}{cc} IL\left(ISO\right) & =-10\log(\frac{P_{A}}{P_{load}})=-10\log\frac{\frac{V_{source}V_{source}^{*}}{8Z_{0}}}{V_{load}I_{load}^{*}}\\ & =-20\log(1+\frac{Z_{CTL}}{2Z_{0}})\\ & =-20\log(1+\frac{R_{CTL}//\frac{1}{j\omega C_{CTL}}}{2Z_{0}})\\ & =-20\log(1+\frac{1}{2Z_{0}(\frac{1}{R_{CTL}}+j\omega C_{CTL})}) \end{array}$$

In Equation (1), *Z*_0_ is the load impedance, and *R*_CTL_ is the resistance of the control element, which was mainly determined by the source-drain resistance (*R*_ds_) at the linear region. *C*_CTL_ is the capacitance of the control element. *P*_A_ is the load power without the control element, and *P*_load_ is the load power with the control element present. Moreover, *r*_g_ is introduced to enhance ISO and reduce signal leakage [12]. *R*_CTL_ can be expressed as follows [13,14]:

(2) $$R_{CTL}=R_{ds}=R_{g}+R_{s}+R_{d}$$

*R*_g_, *R*_s_, and *R*_d_ are the channel resistance, the source-gate, and drain-gate channel resistances, respectively. In the on-state, IL is predominantly influenced by *R*_CTL_, which is the on-state resistance (*R*_on_) of the element.

In Equation (3), *C*_CTL_ can be expressed by the following equation [15]:

(3) $$\begin{array}{cc} C_{CTL} & =C_{ds}//(C_{gd}+C_{gs})\\ & =C_{ds}//\left[\left(C_{gd\_in}+C_{gd\_out})+(C_{gs\_in}+C_{gs\_out}\right)\right] \end{array}$$

where *C*_ds_, *C*_gd_out_/*C*_gs_out_, and *C*_gd_in_/*C*_gs_in_ are the channel capacitance, the parasitic capacitance of gate to drain/source, and internal diode capacitance of gate to drain/source, respectively. For operation well below the switch cutoff frequency, the reactance of the off-state capacitance (*C*_off_) is much greater than *R*_off_, and so *Z*_CTL_ ≈ *C*_CTL_ ≈ *C*_off_.

**Figure 4:**
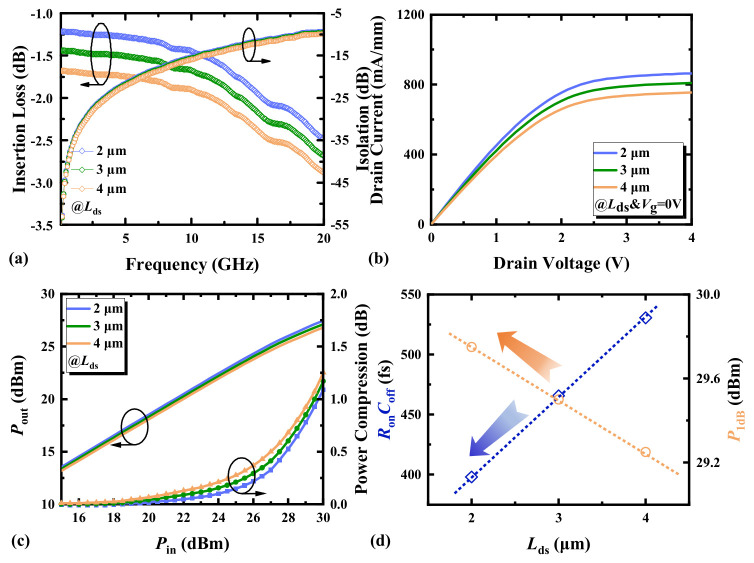
(**a**) The small-signal switching characteristics, (**b**) the output characteristics (*V*_g_ = 0 V), (**c**) the switching power characteristics at 3.6 GHz, and (**d**) the *R*_on_*C*_off_ and *P*_1dB_ at 3.6 GHz of AlGaN/GaN HEMTs with different source-drain spacings.

**Figure 5:**
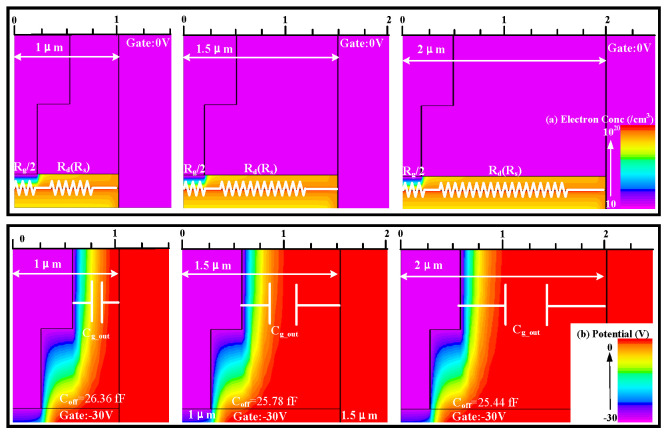
(**a**) The electron concentration distribution of switching devices with different source-drain spacings in the on-state (*V*_g_ = 0 V). (**b**) The potential of switching devices with different source-drain spacings in the off-state (*V*_g_ = −30 V).

Figure 4a demonstrates a situation where the IL and ISO of the switching device both exhibit an increasing trend with the increase in *L*_ds_. From the simulation analysis in Figure 5, it can be seen that an increase in *L*_ds_ leads to an increase in *R*_d_ and *R*_s_, resulting in the degradation of IL. In addition, increasing *L*_ds_ results in the decrease in capacitances *C*_gd_out_ and *C*_gs_out_, while *C*_ds_ remains unchanged; thus, *C*_off_ is reduced. This results in a slight improvement in ISO. Figure 4b indicates that increasing the source-drain spacing and, consequently, increasing the channel length forces the current to travel through a longer path, which may reduce the current density in the channel and affect power output. The switching power characteristics of HEMTs with different source-drain spacings are shown in Figure 4c. Figure 4d shows the influence of the variation in *L*_ds_ of HEMTs on *R*_on_*C*_off_ and *P*_1dB_. The variation in *L*_ds_ has a greater impact on *R*_on_ than on *C*_off_, causing *R*_on_*C*_off_ to increase as *L*_ds_ increases. Therefore, to improve the RF switching performance of HEMTs, the reduction in *L*_ds_ can thereby reduce *R*_on_*C*_off_. Reducing *L*_ds_ can enhance the power handling capability of HEMTs, resulting in the improvement of the power compression of the large signal.

### 3.2. Gate Foot Length

**Figure 6:**
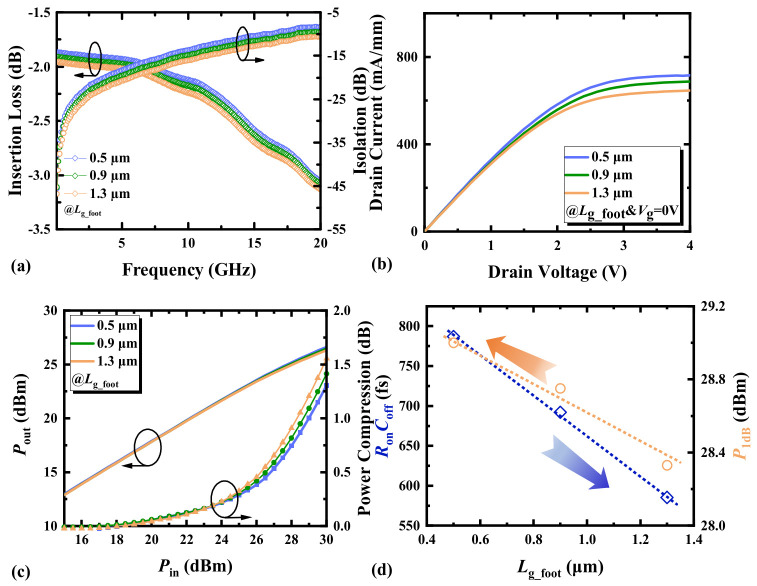
(**a**) The small-signal switching characteristics, (**b**) the output characteristics (*V*_g_ = 0 V), (**c**) the switching power characteristics at 3.6 GHz, and (**d**) the *R*_on_*C*_off_ and *P*_1dB_ at 3.6 GHz of AlGaN/GaN HEMTs with different gate foot lengths.

**Figure 7:**
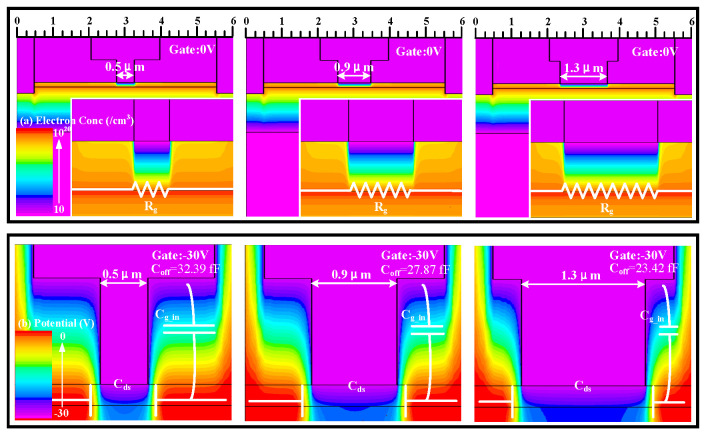
(**a**) The electron concentration distribution of switching devices with different gate foot lengths in the on-state (*V*_g_ = 0 V). (**b**) The potential of switching devices with different gate foot lengths in the off-state (*V*_g_ = −30 V).

The gate foot lengths of the HEMTs in this paper are all shorter than the gate cap lengths. Figure 6a indicates that as *L*_g_foot_ increases, both IL and ISO of the RF switch device increase. As shown in Figure 7, the change in gate foot length causes variations in the depletion region length due to the Schottky contact beneath the gate, where *R*_g_ is positively correlated with *L*_g_foot_, and the influence of *R*_g_ is much greater than that of *R*_d_ and *R*_s_, thereby leading to a change in *R*_on_ along with the trend of *R*_g_ variations. When HEMTs are in the off-state, an increase in *L*_g_foot_ leads to an increase in the equivalent channel capacitance spacing and a decrease in the parasitic capacitance area of the gate cap. As a result, the *C*_ds_ and the *C*_gs_in_/*C*_gd_in_ decrease and ISO improves.

As indicated in Figure 6b, an increase in *L*_g_foot_ results in a reduction in the current density of the switch and a shift in the knee voltage toward the negative direction. As *L*_g_foot_ increases, the knee voltage shifts towards more negative values due to alterations in the internal electric field distribution and carrier transport properties, resulting in reduced current density and affecting the point where current saturation begins, thus modifying the knee voltage [16]. Consequently, in Figure 6c, with an increase in *L*_g_foot_ of the HEMTs, the switch enters the compression phase at an earlier stage. Figure 6d shows the influence of the variation in *L*_g_foot_ of HEMTs on *R*_on_*C*_off_. The influence of *L*_g_foot_ on *C*_off_ is greater than that on *R*_on_. To obtain a better *R*_on_*C*_off_ product and stronger gate control capability, *L*_g_foot_ can be appropriately increased. It also reveals that the trends for selecting *L*_g_foot_ to achieve a low *R*_on_*C*_off_ and a high *P*_1dB_ are opposite. Selecting an appropriate *L*_g_foot_ is beneficial for balancing the small signal performance and power handling of the switch device. This opposing trend clearly demonstrates that *R*_on_*C*_off_, as a small-signal figure of merit, exhibits an inherent conflict between its optimization direction and the requirements for optimizing large-signal power performance. In the design of RF GaN HEMTs, a deliberate trade-off between these aspects must be made based on the target application. Consequently, *R*_on_*C*_off_ alone cannot be used to characterize or predict a device’s large-signal power handling capability.

### 3.3. Gate Cap Length

**Figure 8:**
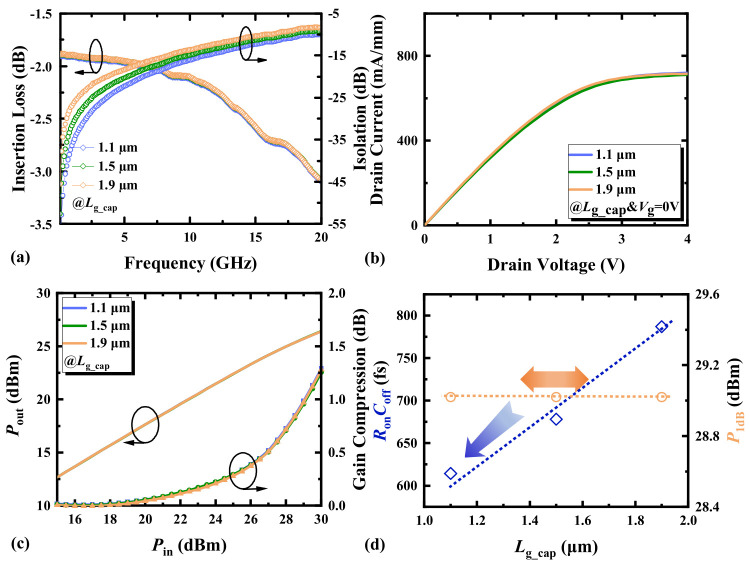
(**a**) The small-signal switching characteristics, (**b**) the output characteristics (*V*_g_ = 0 V), (**c**) the switching power characteristics at 3.6 GHz, and (**d**) the *R*_on_*C*_off_ and *P*_1dB_ at 3.6 GHz of AlGaN/GaN HEMTs with different gate cap lengths.

**Figure 9:**
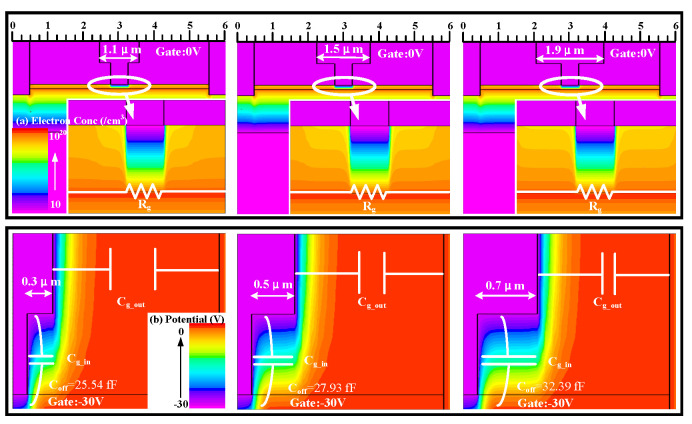
(**a**) The electron concentration distribution of switching devices with different gate foot lengths in the on-state (*V*_g_ = 0 V). (**b**) The potential of switching devices with different gate cap lengths in the off-state (*V*_g_ = −30 V).

From Figure 8a, it can be observed that changing *L*_g_cap_ has little effect on the HEMTs’ IL. Figure 9 indicates that *L*_g_cap_ has a minimal impact on the depletion region formed by Schottky contact, resulting in an insignificant change in the electron concentration under the gate and an insignificant change in *R*_g_. However, altering *L*_g_cap_ causes a significant change in ISO due to a substantial change in *C*_off_ in the off-state. Through the analysis of the potential in the off-state of the device, an increase in *L*_g_cap_ affects the capacitance *C*_gs_in_/*C*_gd_in_ formed between the gate cap and the channel charge [17]. As indicated in Figure 9, this capacitance can be approximated as a parallel plate capacitor, where an increase in *L*_g_cap_ increases the parallel plate area, leading to an increase in *C*_gs_in_/*C*_gd_in_ according to the capacitance formula. On the other hand, an increase in *L*_g_cap_ also reduces the distance between the gate cap and the source/drain ohmic contacts, which are the two plates, thus increasing the parasitic capacitance *C*_gd_out_/*C*_gs_in_. This results in an overall increase in *C*_off_, leading to a degradation in device ISO as *L*_g_cap_ increases. Figure 8b,c indicates that the impact of different *L*_g_cap_ on the saturation current and power compression capability of HEMTs is not significantly pronounced. Figure 8d shows the influence of the variation in *L*_g_cap_ of HEMTs on *R*_on_*C*_off_. Appropriately decreasing *L*_g_cap_ can assist HEMTs in attaining a smaller *R*_on_*C*_off_.

### 3.4. Gate Width

**Figure 10:**
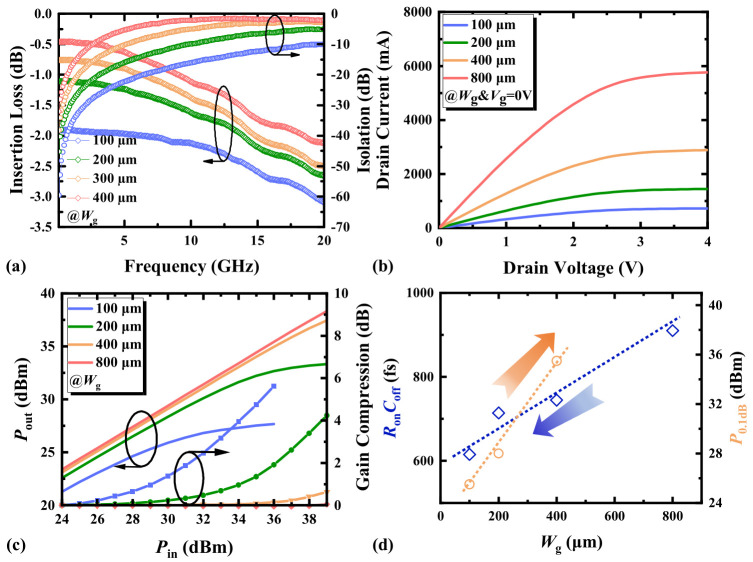
(**a**) The small-signal switching characteristics, (**b**) the output characteristics (*V*_g_ = 0 V), (**c**) the switching power characteristics at 3.6 GHz, and (**d**) the *R*_on_*C*_off_ and *P*_1dB_ at 3.6 GHz of AlGaN/GaN HEMTs with different gate widths.

Figure 10a demonstrates the study of IL and ISO with different gate widths. The research on the impact of *W*_g_ is relatively straightforward. As *W*_g_ increases, the cross-sectional area of the 2DEG channel also increases, allowing more electrons to participate in conduction, thereby reducing the channel resistances *R*_g_, *R*_d_, and *R*_s_. The reduction in channel resistance can decrease the resistive loss of the device, thereby reducing IL. The relationship between resistive loss, channel resistance, and current is given by the following [18]:

(4) $$P_{loss}\propto R_{ds}\cdot I^{2}$$

In the off-state, a wider gate increases the overlap area between the gate and drain/source, leading to an increased *C*_gd_/*C*_gs_. This capacitance may induce additional parasitic currents, thereby degrading ISO between the input and output ports. Moreover, increasing *W*_g_ expands the depletion region, which enhances the internal inherent capacitances and consequently increases *C*_ds_. Overall, the *C*_off_ increases significantly, leading to degraded ISO.

As depicted in Figure 10b, the expansion of the effective conductive area with increasing *W*_g_ reduces the on-resistance, thereby improving the device’s overcurrent capability. A reduced on-resistance minimizes conduction losses in the on-state, while enhanced overcurrent capability enables higher power-handling capacity in the device [19]. As shown in Figure 10c, HEMTs with larger *W_g_* exhibit higher power compression capabilities, making them more widely applicable in high-power RF modules. Figure 10d indicates that the trends for selecting *W*_g_ to achieve a low *R*_on_*C*_off_ and a high *P*_0.1dB_ are opposite. The influence of *W*_g_ on *C*_off_ is greater than that on *R*_on_. Therefore, *R*_on_*C*_off_ increases as *W*_g_ increases. From the perspective of *W*_g_ design, there exists an inherent trade-off between improving power tolerance and *R*_on_*C*_off_ performance.

### 3.5. Gate Bias Resistance

**Figure 11:**
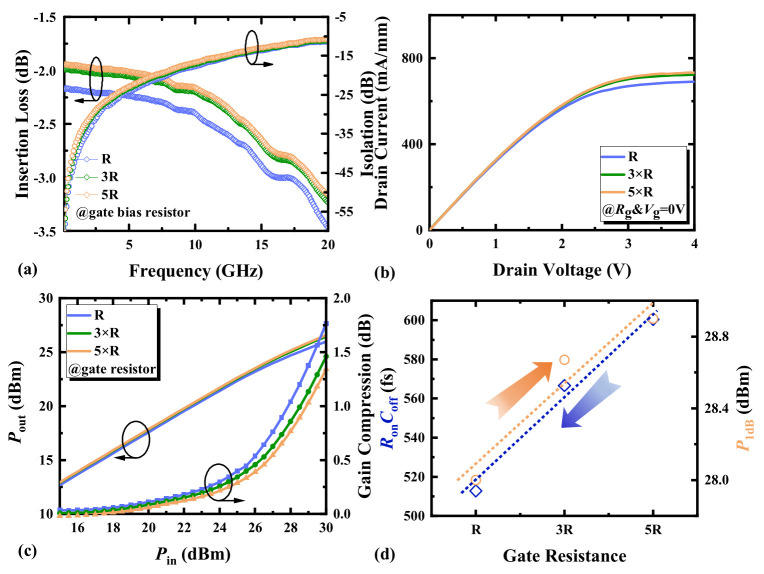
(**a**) The small-signal switching characteristics, (**b**) the output characteristics (*V*_g_ = 0 V), (**c**) the switching power characteristics at 3.6 GHz, and (**d**) the *R*_on_*C*_off_ and *P*_1dB_ at 3.6 GHz of AlGaN/GaN HEMTs with different gate bias resistances.

Figure 11a focuses on the small-signal characteristics of HEMTs with different *r*_g_. As *r*_g_ increases, IL is optimized at the expense of ISO degradation. The RF switch device’s Y-parameters are as follows [20,21]:

(5) $$Y_{ctl}=\left[\begin{array}{l} y_{11}\\ y_{21} \end{array}\right.\left.\begin{array}{l} y_{12}\\ y_{22} \end{array}\right]$$

(6) $$\begin{array}{ll} y_{11} & =j\omega C_{ds}+\frac{1}{R_{ds}}+\frac{1}{2r_{g}+\frac{1}{j\omega C_{g}}}+\frac{1}{\frac{2}{j\omega C_{g}}-\frac{1}{\omega^{2}{C_{g}}^{2}r_{g}}}\\ & =y_{22} \end{array}$$

(7) $$\begin{array}{ll} y_{12} & =-j\omega C_{ds}-\frac{1}{R_{ds}}-\frac{1}{\frac{2}{j\omega C_{g}}-\frac{1}{\omega^{2}{C_{g}}^{2}r_{g}}}\\ & =y_{21} \end{array}$$

By converting Y-parameters to S-parameters, *S*_21_ can be expressed as follows [11,22]:

(8) $$S_{21}\propto \frac{1}{\Gamma }$$

(9) $$\begin{array}{ll} \frac{1}{\Gamma } & =\frac{(Z_{ctl}+Z_{0})+Z_{0}}{(Z_{ctl}+Z_{0})-Z_{0}}\\ & =1+\frac{2Z_{0}}{Z_{ctl}}\\ & =1+2Z_{0}\cdot Y_{ctl} \end{array}$$

The equivalent circuit of RF switch devices can be represented by *Y*_ctl_. In Equations (5)–(9), analysis of the admittance matrix elements *y*_11_ and *y*_22_ demonstrates that increasing *r*_g_ enhances the *C*_g_-to-ground decoupling. This improved decoupling effect reduces port-ground reflections, consequently increasing the magnitude of *S*_21_. This indicates that an increase in *r*_g_ will reduce IL to some extent. In the off-state, increasing *r*_g_ enhances the C_g_-to-ground decoupling effect, thereby diminishing gate-channel control efficacy. The underlying mechanism involves the substantial gate resistance generating an additional voltage drop that causes the effective gate potential at the Schottky interface to become more positive relative to the external DC bias. This potential shift results in a measurable degradation of ISO.

As observed from Figure 11b, for RF switch devices with gate biased at 0 V, a smaller *r*_g_ weakens the *C*_g_-to-ground decoupling effect. Consequently, this enhances gate control capability, accelerating current saturation while reducing channel current density. Such current density degradation ultimately compromises power output performance. As shown in Figure 11c, when *r*_g_ increases, the fluctuations in gate voltage increase, leading to intensified nonlinear behavior of the device and more severe power compression. It is known that increasing *r*_g_ reduces IL, and a larger *r*_g_ can reduce power leakage through the gate, thereby decreasing losses. As *r*_g_ continues to increase, these differences diminish. Figure 11d shows the influence of the variation in *r*_g_ of HEMTs on *R*_on_*C*_off_ and *P*_1dB_. Increasing *r*_g_ will improve the power handling capability of HEMTs, but it will deteriorate *R*_on_*C*_off_. When selecting bias resistance, it is necessary to balance *R*_on_*C*_off_ and power compression.

### 3.6. Gate Metal Work Function

**Figure 12:**
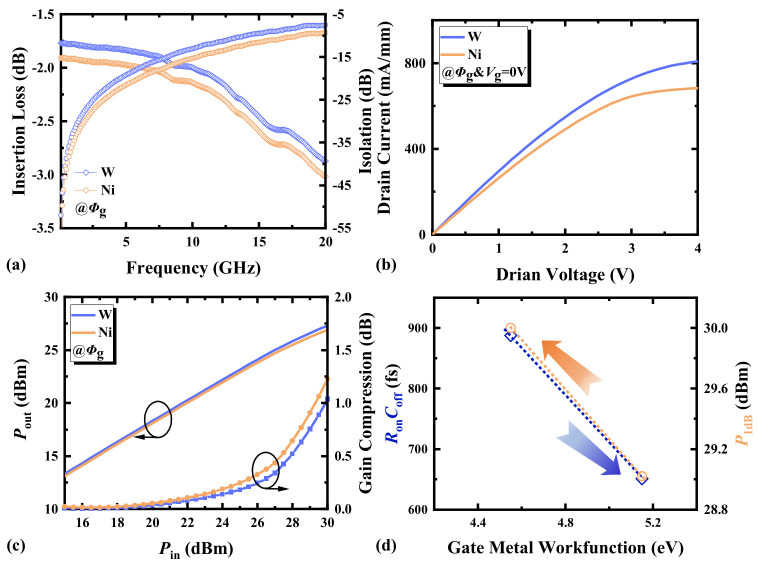
(**a**) The small-signal switching characteristics, (**b**) the output characteristics (*V*_g_ = 0 V), (**c**) the switching power characteristics at 3.6 GHz, and (**d**) the *R*_on_*C*_off_ and *P*_1 dB_ at 3.6 GHz of AlGaN/GaN HEMTs with different gate metal work functions.

Figure 12a shows the small-signal switching characteristics with different *Φ*_g_. The two metals used for forming the Schottky contact gates are tungsten (W) and nickel (Ni). The work function of W is 4.55 eV, while that of Ni is 5.15 eV. IL of HEMTs with W as the gate metal is lower than that with Ni, but its ISO is worse. *Φ*_g_ directly affects the charge distribution and control capability between the gate and the channel [23,24]. As shown in Figure 12b,c, higher *Φ*_g_ can lead to the early saturation of electron velocity in the channel, resulting in a lower current density and faster power compression.

**Figure 13:**
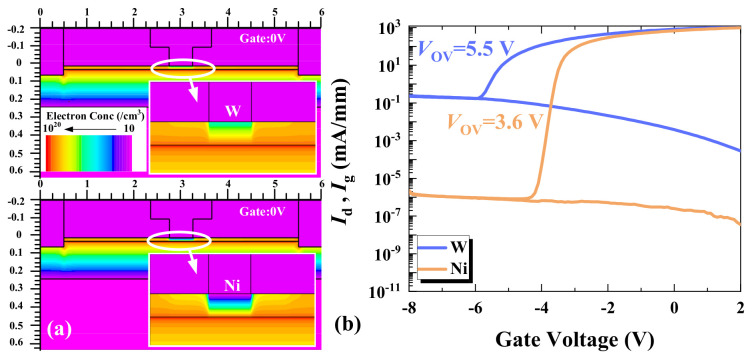
The electron concentration distribution of switching devices with different *Φ*g in the (**a**) on-state (*V*_g_ = 0 V). (**b**) Transfer characteristics of switching devices with different *Φ*_g_ at *V*_DS_ = 10 V.

From the simulation and measurement results shown in Figure 13, it is evident that when the gate bias is 0 V, HEMTs operate in the on-state, and the electron concentration under the W gate is significantly higher than that under the Ni gate. Different *Φ*_g_ results in different threshold voltages (*V*th). At *V*_g_ = 0 V, this leads to distinct overdrive voltages (*V*_ov_ = *V*_g_ − *V*th) and different saturation current densities. Theoretically, the lower work function of W yields a more negative *V*th, higher *V*_ov_, larger drain current density, better IL, and higher *P*_1 dB_ at *V*_g_ = 0 V. Conversely, at *V*_g_ = −30 V, the reduced negative *V*_ov_ degrades ISO.

Figure 12d illustrates the impact of *Φ*_g_ variation on *R*_on_*C*_off_ and *P*_1 dB_ in HEMT devices. Increasing *Φ*_g_ can reduce the product of *R*_on_*C*_off_, but it will lower *P*_1 dB_. The appropriate *Φ*_g_ is beneficial for balancing low *R*_on_*C*_off_ and high power-handling capability. These statements clarify that the influence patterns of the work function revealed in this study were characterized under the specific applied DC bias conditions. The conclusions directly reflect the core mechanism by which the work function difference alters the threshold voltage through flat-band voltage modulation near this specific operating point.

**Figure 14:**
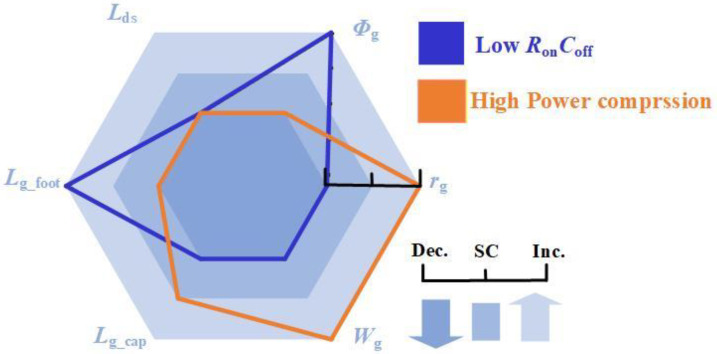
Selection of device structures for low *R*_on_*C*_off_ and high power compression. Decrease (Dec.). Slight change (SC). Increase (Inc.).

Figure 14 presents the design principle for low *R*_on_*C*_off_ and high power compression. As shown in Figure 14, when selecting the values of *L*_g_foot_, *W*_g_, *r*_g_, and *Φ*_g_ for HEMTs, a trade-off needs to be made between low *R*_on_*C*_off_ and high power compression. Subsequent optimization of the gate structure and selection of appropriate gate metals enabled the tailoring of AlGaN/GaN HEMTs for diverse RF switch applications.

## 4. Conclusions

In this paper, we investigated the structure-dependent parameter trade-off optimization on *R*_on_*C*_off_ and power compression of AlGaN/GaN HEMTs for RF switch application. It was found that *R*_on_*C*_off_ can be effectively reduced by increasing *L*_g_foot_, decreasing *L*_g_cap_, reducing *r*_g_, and adopting a high *Φ*_g_. But these parameter modifications exhibit antagonistic effects on power compression and *R*_on_*C*_off_. These results provide design guidelines for RF switch devices operating in diverse application environments.

## Figures and Tables

**Table 1:** Device parameter setting.

No.	*L*_ds_/μm	*L*_g_foot_/μm	*L*_g_cap_/μm	*W*_g_/μm	*r*_g_	Schottky Contact Gate Metal
A	2/3/4	0.5	1.1	100	7 × R	Ni
B	5	0.5/0.9/1.3	1.9	100	7 × R	Ni
C	5	0.5	1.1/1.5/1.9	100	7 × R	Ni
D	5	0.5	1.1	100/200/400/800	7 × R	Ni
E	5	0.5	1.1	100	R/3 × R/5 × R	Ni
F	5	0.5	1.3	100	7 × R	Ni/W

Note: *L*_ds_ is the source-drain spacing. *L*_g_foot_ is the gate foot length. *L*_g_cap_ is the gate cap length. *W*_g_ is the gate width. R = 2000 Ω.

## Data Availability

The original contributions presented in this study are included in the article. Further inquiries can be directed to the corresponding author.
